# Association between chronic kidney disease and mortality in patients with a confirmed COVID-19 diagnosis

**DOI:** 10.7717/peerj.13437

**Published:** 2022-06-14

**Authors:** Jacqueline Betsabe Puicón-Suárez, Sandra Zeña-Ñañez, Virgilio E. Failoc-Rojas

**Affiliations:** 1 Sociedad Científica de Estudiantes de Medicina, SOCIEM-UNPRG, Universidad Nacional Pedro Ruiz Gallo, Lambayeque, Perú; 2 Universidad Continental, Huancayo, Perú; 3 Universidad San Ignacio de Loyola, Lima, Peru; 4 EsSalud, Instituto de Evaluación de Tecnologías en Salud e Investigación, Lima, Lima, Peru

**Keywords:** COVID-19, Chronic kidney disease, Mortality, Mexico

## Abstract

**Objective:**

To determine the association between chronic kidney disease (CKD) and mortality in persons with a confirmed coronavirus disease 2019 (COVID-19) diagnosis.

**Methods:**

Cross-sectional secondary baseline study. The study population consisted of 243,065 patients confirmed to have COVID-19 during May–December 2020. Stata 16.0 was used for statistical analysis, Chi-square test was used for bivariate analysis, and Poisson regression with robust variances was used for multiple analysis.

**Results:**

The prevalence of patients with a confirmed COVID-19 diagnosis who had CKD and died was 1.42 times the prevalence of mortality in those without CKD. The comorbidities combined with CKD that presented the highest probability of mortality were diabetes mellitus and hypertension.

**Conclusions:**

CKD is associated with a high mortality rate in patients with a confirmed COVID-19 diagnosis. Patients with CKD, diabetes mellitus, and arterial hypertension have a higher prevalence of mortality than those without comorbidities.

## Introduction

The physiopathology of coronavirus disease 2019 (COVID-19) involves inflammatory processes that initially occur as a local immune response triggered by the destruction of lung cells (Tay et al., 2020). However, the macrophage-mediated response is deregulated by the severe acute respiratory syndrome-coronavirus-2 (SARS-CoV-2) (Merad & Martin, 2020; Tay et al., 2020; Yuki, Fujiogi & Koutsogiannaki, 2020), producing a hyperinflammatory response (Callender et al., 2020; Merad & Martin, 2020). Among the multi-systemic effects of COVID-19 are the kidney manifestations that lead to severe and/or fatal results (Adapa et al., 2020; D’Marco et al., 2020; Kunutsor & Laukkanen, 2020).

Patients with chronic kidney disease (CKD) experience alterations, such as chronic inflammation, enhanced oxidative stress (Lepe-Zúñiga, Morales-Molina & García-Nandayapa, 2016), and epigenetic modifications of hematopoietic stem cells (Betjes, Meijers & Litjens, 2013; Syed-Ahmed & Narayanan, 2019), in the immune system. These changes result in a loss of immune function, especially lymphocyte function (Betjes, Meijers & Litjens, 2013; Syed-Ahmed & Narayanan, 2019), which increases the risk of contracting infections such as pneumonia (Callender et al., 2020; Lepe-Zúñiga, Morales-Molina & García-Nandayapa, 2016; Liu et al., 2015).

The systematic reviews and meta-analysis by Singh et al. (2020) and Nandy et al. (2020) found a higher risk of mortality and severity of COVID-19 (odds ratio (OR) 3.86 and 5.32, respectively) in those who had CKD compared to those who did not have CKD. The meta-analysis by Henry & Lippi (2020) that included four studies conducted in Chinese population with COVID-19 until March 2020 found that the risk of developing fever was three times higher in patients with CKD than in those without CKD (OR: 3.03; 95% CI [1.09–8.47]).

According to the above findings, CKD is related to COVID-19 severity and mortality risk (D’Marco et al., 2020). However, 74% of the studies were performed in China and do not provide data on the Latin American scenario. Moreover, some meta-analyses show moderate-to-high heterogeneity (Adapa et al., 2020; Henry & Lippi, 2020; Nandy et al., 2020), it has a mortality rate of 23 per 100 thousand inhabitants nationwide and during three consecutive years (2016, 2017 and 2018) about 516,287 dialysis procedures were recorded as a cumulative value (Dorantes-Carrillo et al., 2021); it is important to perform a study that allows to understand the prevalence of mortality in patients with CKD. Based on the objective results, it would be possible to propose health strategies or policies that promote measures enabling greater attention to CKD, a preventable and treatable disease. Likewise, the results would provide evidence for an inefficient healthcare capacity that compromises the correct medical attention to older adults with CKD and COVID-19. This study aimed to determine the association between CKD and mortality in patients with a confirmed COVID-19 diagnosis.

## Methods

### Design and study population

A cross-sectional study of secondary analysis of the Mexican open COVID-19 database was performed. The information in this database is collected by the Epidemiological Surveillance System for Viral Respiratory Diseases (SISVER) (Datos.gob.mx, 2020).

The database population was from Mexico, a country with territories in both North America and Central America (Programa de las Naciones Unidas para el Desarrollo, 2020). With a total population of 127,575,529 inhabitants, Mexico ranks as the 10^th^ most populous country in the world and the third most populous in the Americas (Grupo Banco Mundial, 2019). Totally, 2,977,002 individuals were included in the database, representing 2.33% of the country’s total population. The SISVER included both outpatients and inpatients with symptoms of viral respiratory disease and suspected to have SARS-CoV-2 (Datos.gob.mx, 2020).

The study population consisted of 1,156,770 patients with a confirmed diagnosis of COVID-19 during the months of May–December 2021. A confirmed COVID-19 diagnosis case was defined by the following: the epidemiological clinical association (applicable when the case was reported to be a contact of a SARS-CoV-2 positive person and was registered in the SISVER), the ruling committee (only applies to deaths where the patient was not sampled or a sample was taken but was invalid), presence of a laboratory sample or antigenic test positive for SARS-CoV-2. The entire sampling frame was used.

**Figure 1:**
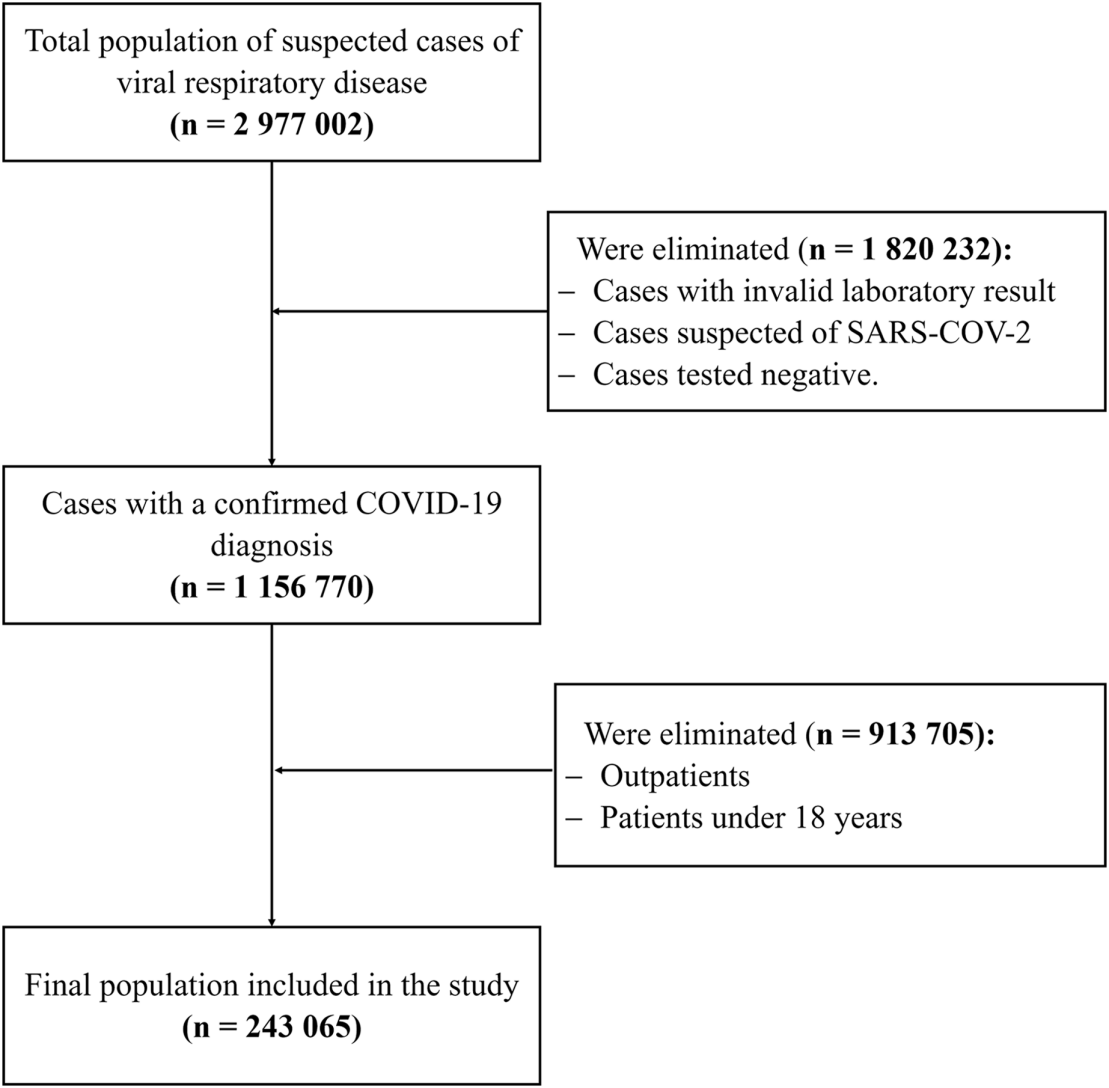
Study population selection flowchart in patients with COVID-19. This flow chart shows the number of patients selected according to inclusion and exclusion criteria who finally entered the analysis.

Inclusion criteria included patients with a confirmed diagnosis of COVID-19, patients aged 18 years or older and who had been hospitalized. Cases with invalid laboratory result, cases suspected of SARS-CoV-2, Cases tested negative and outpatients were excluded (See Fig. 1).

The statistical software Stata® 16.0 was used to calculate the statistical power for the two proportions. The patient populations with and without CKD were compared. According to the study by Parra-Bracamonte, Lopez-Villalobos & Parra-Bracamonte (2020), the expected prevalence was 39% in patients with COVID-19 who had CKD and died, and it was 19.5% in patients with COVID-19 who did not have CKD and died. Finally, using a 95% significance level, a power of 100% was obtained.

### Definition of variables

The independent variable, CKD, is defined as the presence of structural or functional renal anomalies that persists for more than 3 months, with or without impaired renal function, or a glomerular filtration rate of <60 mL/min/1.73 m^2^ without other signs of kidney disease (CENETEC México, 2010; Sellarés, 2019). In this study, the CKD variable was considered dichotomous (yes/no).

The dependent variable was mortality, defined as the death of the patient according to the Intensive Care Unit (ICU) database. The covariates were age, sex, and smoking as well as diagnoses of pneumonia, chronic obstructive pulmonary disease (COPD), obesity, diabetes mellitus (DM), arterial hypertension (HT), cardiovascular disease, and immunosuppression.

### Procedures

For collecting information, the database was provided by SISVER, a system executed by 475 monitoring units of the entire viral respiratory disease health sector (USMER) in Mexico (datos.gob.mx). Health personnel used the epidemiological study file of a suspected case of viral respiratory disease that contains the record of personal and clinical data, treatment, epidemiological history, laboratory results, and case evolution. The data were set up and updated on a regular basis, being able to freely access their registry through the open data catalog of the Government of the Mexican Republic. The updated database through 05 December 2020, was downloaded and imported in a “comma-separated values” (.csv) format to the statistical software Stata 16.0. Dates-associated variables were imported as a string (.str). Data cleaning was performed, and the confirmed COVID-19 cases were selected. Data of patients without confirmed COVID-19 were removed from the database. It was verified that there were no duplicate or lost data in the database.

### Statistical analysis

Stata® version 16.0 (College Station, TX, USA) was used for the statistical analysis. The categorical variables were expressed using absolute and relative frequencies. The Chi-square test was used for the bivariate analysis comparing the proportions. The multiple analysis considered mortality and ICU admission as the outcome variables. This analysis used a generalized linear model with Poisson family, logarithmic link, and robust variances to estimate the prevalence ratio (PR). The CI was 95%, and *p* < 0.05 was considered statistically significant, in the case of multiple comparisons, the Bonferroni correction was considered. A crude and adjusted analysis of the presence of CKD with another comorbidity was performed to know the effect on the prevalence of mortality. PR was used to assess the effects of CKD, DM, HT, COPD, and their combinations compared with no comorbidity, generated as individual models. Crude and adjusted PRs were estimated for age, sex, COPD, obesity, diabetes mellitus, hypertension and immunosuppression as well as for their 95% CI.

### Ethical aspects

The database is available in the open data catalog of the Government of the Mexican Republic (https://datos.gob.mx/) according to the decree published on 20 February 2015 in the Official Gazette of the Federation, which concluded that the general population is able to access it (Diario Oficial de la Federación, 2015).

Open database presents a coded identification of the participants, which cannot be accessed. Likewise, there is no personal information of the patients that provides details of their identification. Furthermore, there was no contact with human beings, and, therefore, the study did not require the approval of an ethics committee. However, during the implementation of the study, the ethical principles of the Declaration of Helsinki were followed.

## Results

This study included 1,156,770 cases with a confirmed COVID-19 diagnosis out of a total of 2,977,002 suspected cases of viral respiratory disease. The rest of the population had an invalid laboratory result, were suspected of SARS-CoV-2, or were tested negative. As per the selection criteria, outpatients and those <18 years of age were excluded, which resulted in a final population of 243,065 cases with a confirmed COVID-19 diagnosis.

The age range with the highest frequency of confirmed COVID-19 diagnosis was 46–65 years, followed by the population of >65 years of age (45.94% and 32.46%, respectively). The confirmed COVID-19 diagnosis was more frequent in men than in women, with an approximate ratio of 3:2 (60.35% and 39.65% respectively).

Hypertension, DM, and obesity were the comorbidities with the highest frequency (38.18%, 33.18%, and 22.73% respectively), occurring in more than 90% of the population with a confirmed COVID-19 diagnosis. Approximately two thirds of the cases presented pneumonia as a complication, and 8.46% of the patients were admitted to the ICU. Regarding CKD, it was found that 5.37% of the population had this disease (See Table 1).

**Table 1:** Characteristics of patients diagnosed with coronavirus disease 2019 (COVID-19) in Mexico.

Characteristics	COVID-19	
	*n*	%
Age (years)		
18–45	52,495	21.60
46–65	111,667	45.94
>65	78,903	32.46
Sex		
Female	96,365	39.65
Male	146,700	60.35
Smoking^°^		
Yes	18,910	7.78
No	223,132	91.80
Pneumonia		
Yes	157,549	64.82
No	85,516	35.18
COPD^°^		
Yes	8,903	3.66
No	233,180	95.93
Obesity^°^		
Yes	55,237	22.73
No	186,819	76.86
Diabetes mellitus^°^		
Yes	80,653	33.18
No	161,276	66.35
Arterial hypertension^°^		
Yes	92,811	38.18
No	149,191	61.38
Cardiovascular disease^°^		
Yes	10,405	4.28
No	231,613	95.29
Immunosuppression^°^		
Yes	5,182	2.13
No	236,837	97.44
Chronic kidney disease^°^		
Yes	13,062	5.37
No	229,009	94.22
ICU^°^		
Yes	20,556	8.46
No	221,881	91.28
Died		
Yes	96,916	39.87
No	146,149	60.13

**Notes:**

° Some variables may have missing data.

COPD, Chronic Obstructive Pulmonary Disease.

ICU, Intensive Care Unit.

Regarding mortality in Mexican population with a confirmed COVID-19 diagnosis, the number of patients >65 years of age who died was greater than the cases in the age group of 46–65 years (55.62% *vs* 38.37%, *p* < 0.001). Considering the Bonferroni correction, this comparison remains statistically significant. Patients with CKD and COVID-19 experienced higher mortality than those without CKD (55.29% *vs* 38.95%). CKD was the comorbidity with a higher frequency of mortality than other diseases (See Table 2).

**Table 2:** Bivariate analysis of the characteristics of patients with coronavirus disease 2019 disease (COVID-19) in Mexico.

	COVID-19									
Characteristics	Died		Did not die			Admitted to ICU		Not admitted to ICU		
	*n*	%	*n*	%	*p* value	*n*	%	*n*	%	*p* value
Age* (years)					<0.001					<0.001
18–45	10,182	19.40	42,313	80.60		4,151	7.92	48,244	92.08	
46–65	42,852	38.37	68,815	61.63		9,729	8.73	101,654	91.27	
Over 65	43,882	55.62	35,021	44.38		6,676	8.49	71,983	91.51	
Sex*					<0.001					<0.001
Female	35,388	36.72	60,977	63.28		7,375	7.67	88,754	92.33	
Male	61,528	41.94	85,172	58.06		13,181	9.01	133,127	90.99	
Smoking*					<0.001					0.550
Yes	7,864	41.61	11,036	58.39		1,570	8.32	17,295	91.68	
No	88,529	39.68	134,603	60.32		18,801	8.45	203,739	91.55	
Pneumonia*					<0.001					<0.001
Yes	72,643	46.11	84,906	53.89		17,982	11.43	139,322	88.57	
No	24,273	28.38	61,243	71.62		2,574	3.02	82,559	96.98	
COPD*					<0.001					0.207
Yes	4,646	52.18	4,257	47.82		716	8.07	8,154	91.93	
No	91,768	39.36	141,412	60.64		19,657	8.45	212,929	91.55	
Obesity*					<0.001					<0.001
Yes	22,899	41.46	32,338	58.54		5,855	10.63	49,211	89.37	
No	73,524	39.36	113,295	60.64		14,519	7.79	171,844	92.21	
Diabetes mellitus*					<0.001					<0.001
Yes	37,859	46.94	42,794	53.06		7,097	8.82	73,351	91.18	
No	58,505	36.28	102,771	63.72		13,246	8.23	147,609	91.77	
Arterial hypertension*					<0.001					0.455
Yes	44,761	48.23	48,050	51.77		7,852	8.49	84,671	91.51	
No	51,630	34.61	97,561	65.39		12,503	8.40	136,349	91.60	
Cardiovascular disease*					<0.001					<0.001
Yes	5,205	50.02	5,200	49.98		974	9.38	9,408	90.62	
No	91,180	39.37	140,433	60.63		19,390	8.39	211,619	91.61	
Immunosuppression*					<0.001					0.128
Yes	2,384	46.01	2,798	53.99		466	9.02	4,701	90.98	
No	94,008	39.69	142,829	60.31		19,898	8.42	216,327	91.58	
Chronic kidney disease*					<0.001					<0.001
Yes	7,222	55.29	5,840	44.71		822	6.31	12,215	93.69	
No	89,195	38.95	139,814	61.05		19,548	8.56	208,859	91.44	

**Notes:**

* Chi^2^ test was used.

COPD, Chronic Obstructive Pulmonary Disease.

ICU, Intensive Care Unit.

According to the admission to ICU in the Mexican population with a confirmed COVID-19 diagnosis, the number of patients >65 years of age who were admitted to the ICU was similar to those >45 years but <65 years of age (8.49% *vs* 8.73%, *p* < 0.001). Considering the Bonferroni correction, this comparison remains statistically significant. Men with a confirmed COVID-19 diagnosis presented a higher number of admissions to the ICU than women (9.01% *vs* 7.67%, *p* < 0.001), as seen in the mortality frequency. Furthermore, 11.43% patients with pneumonia were admitted to the ICU *vs* 3.02% without pneumonia. Patients with CKD and COVID-19 were admitted to the ICU less frequently than those without CKD (6.31% *vs* 8.56%, *p* < 0.001), while patients with obesity and COVID-19 were admitted to the emergency room more often than those without obesity (10.63% *vs* 7.79%, *p* < 0.001). (See Table 2).

In the Mexican population with confirmed COVID-19, the prevalence of mortality in patients >65 years of age was 2.87 times than that in patients in the age group of 18–45 years (95% CI [2.81–2.92]; *p* < 0.001). The prevalence of mortality in women was 22% (95% CI [22–23]; *p* < 0.001), which was lower than that in men. CKD and hypertension were found to be the most prevalent comorbidities in people who faced mortality (PR 1.42 and 1.39, respectively). The prevalence of confirmed COVID-19 cases that had CKD and died was 1.42 (95% CI [1.40–1.44]; *p* < 0.001) times that of cases with no kidney disease as a comorbidity. This number decreased to 1.29 (95% CI [1.27–1.31]; *p* < 0.001) in the multiple analysis when adjusting for the variables of age, sex, COPD, obesity, diabetes, hypertension, and immunosuppression. In the multiple analysis, it was found that the PR of death for the cases with obesity *vs* no obesity as a comorbidity was 1.10 times (95% CI [1.09–1.12]; *p* < 0.001) higher than that observed in bivariate analysis (PR: 1.05, 95% CI [1.04–1.07]; *p* < 0.001). It stands out that the death prevalence with CKD as a comorbidity was 1.42 (95% CI [1.40–1.44]; *p* < 0.001) times than that without kidney disease. However, this prevalence was reduced to 1.29 (95% CI [1.27–1.31]; *p* < 0.001) times in the multiple analysis (see Table 3).

**Table 3:** Bivariate and multiple analysis of the association between chronic kidney disease and mortality.

	Died						Admitted to ICU					
Characteristics	Bivariate analysis			Multiple analysis*			Bivariate analysis			Multiple analysis*		
	PR	95% CI	*p*	PR	95% CI	*p*	PR	95% CI	*p*	PR	95% CI	*p*
Age (years)			<0.001			<0.001			<0.001			<0.001
18–45	Ref.			Ref.			Ref.			Ref.		
46–65	1.98	[1.94–2.02]		1.90	[1.86–1.93]		1.10	[1.06–1.14]		1.09	[1.06–1.13]	
>65	2.87	[2.81–2.92]		2.72	[2.66–2.77]		1.07	[1.03–1.11]		1.10	[1.06–1.15]	
Sex			<0.001			<0.001			<0.001			<0.001
Male	Ref.			Ref.			Ref.			Ref.		
Female	0.88	[0.87–0.88]		0.85	[0.84–0.85]		0.85	[0.83–0.88]		0.84	[0.81–0.86]	
Smoking			<0.001			–			0.550			–
No	Ref.						Ref.					
Yes	1.05	[1.03–1.07]		–	–		0.99	[0.94–1.03]		–	–	
Pneumonia			<0.001			–			<0.001			–
No	Ref.						Ref.					
Yes	1.62	[1.61–1.64]		–	–		3.78	[3.63–3.94]		–	–	
COPD			<0.001			<0.001			0.208			0.082
No	Ref.			Ref.			Ref.			Ref.		
Yes	1.33	[1.30–1.35]		1.05	[1.03–1.07]		0.96	[0.89–1.03]		0.94	[0.87–1.01]	
Obesity			<0.001			<0.001			<0.001			<0.001
No	Ref.			Ref.			Ref.			Ref.		
Yes	1.05	[1.04–1.07]		1.10	[1.09–1.12]		1.36	[1.33–1.40]		1.38	[1.34–1.42]	
Diabetes mellitus			<0.001			<0.001			<0.001			<0.001
No	Ref.			Ref.			Ref.			Ref.		
Yes	1.29	[1.28–1.31]		1.09	[1.08–1.16]		1.07	[1.04–1.10]		1.07	[1.04–1.11]	
Arterial hypertension			<0.001			<0.001			0.455			0.082
No	Ref.			Ref.			Ref.			Ref.		
Yes	1.39	[1.38–1.41]		1.11	[1.10–1.12]		1.01	[0.98–1.04]		0.97	[0.94–1.00]	
Cardiovascular disease			<0.001			–			<0.001			<0.001
No	Ref.						Ref.					
Yes	1.27	[1.25–1.30]		–	–		1.12	[1.05–1.19]		–	–	
Immunosuppression			<0.001			–			0.127			0.008
No	Ref.						Ref.			Ref.		
Yes	1.16	[1.12–1.19]		–	–		1.07	[0.98–1.17]		1.13	[1.03–1.23]	
Chronic kidney disease			<0.001			<0.001			<0.001			<0.001
No	Ref.			Ref.			Ref.			Ref.		
Yes	1.42	[1.40–1.44]		1.29	[1.27–1.31]		0.74	[0.69–0.79]		0.73	[0.68–0.78]	

**Notes:**

* Adjusted for the variables: age, sex, COPD, obesity, diabetes mellitus, hypertension and immunosuppression.

PR, Prevalence Ratio; 95% CI, 95% Confidence Interval.

COPD, Chronic Obstructive Pulmonary Disease.

ICU, Intensive Care Unit.

In the association between CKD and admission to ICU in the Mexican population with a confirmed COVID-19 diagnosis, the prevalence of mortality in the age group of 45–65 years was 1.10 times than that in the age group of 18–45 years (95% CI [1.06–1.14]; *p* < 0.001). Multiple analysis adjusted for the variables of age, sex, COPD, obesity, diabetes, hypertension, and immunosuppression showed that the prevalence of being >65 years of age and admitted to the ICU was 1.10 times than that of being >45 years of age and admitted to the ICU (95% CI [1.06–1.15]; *p* < 0.001). The prevalence of admission to the ICU with CKD was 26% (95% CI [21–31]; *p* < 0.001) lower than that of admission to the ICU with no CKD (see Table 3).

According to the risk of dying with CKD and other diseases in the Mexican population with confirmed COVID-19 diagnosis, the comorbidities combined with CKD that presented a similar probability of dying were DM and hypertension. In Mexicans with a confirmed COVID-19 diagnosis, the prevalence of mortality in those with CKD and DM was 1.64 (95% CI [1.61–1.67]; *p* < 0.001) times than that in patients without any comorbidity. The prevalence of mortality in patients with CKD and hypertension was 1.65 (95% CI [1.62–1.69]; *p* < 0.001) times than that in patients without any comorbidity (See Table 4).

**Table 4:** Bivariate analysis of risk of death in chronic kidney disease and other diseases.

Characteristic	Mortality		
	PR*	95% CI	*p*
No sickness^&^			<0.001
CKD	1.40	[1.33–1.47]	
No sickness^&^			<0.001
CKD + DM	1.64	[1.61–1.67]	
No sickness^&^			<0.001
CKD + HT	1.65	[1.62–1.69]	
No sickness^&^			<0.001
CKD + DM + HT	1.82	[1.78–1.86]	

**Notes:**

* PR, Prevalence Ratio; 95% CI, 95% Confidence Interval.

CKD, Chronic Kidney Disease; DM, Diabetes Mellitus; HT, Arterial hypertension.

& The analyses in the table correspond to different models.

## Discussion

In our study we found that having had COVID-19 and CKD presented a high mortality in the Mexican population with a confirmed COVID-19 diagnosis, and that this comorbidity is an important risk factor for death (PR: 1.42, 95% CI [1.40–1.44]).

CKD is an important risk factor for mortality in COVID-19. Previous reports in Mexican patients with confirmed COVID-19 showed frequencies of CKD presentation ranging from 2.4% to 4.78%, while in this study the frequency of presentation rose to 5.37% (Bello-Chavolla et al., 2020; Hernández-Galdamez et al., 2020). This difference could be the result of the number of patients in the hospitalized population of the cited investigations, they represent 8.20% and 9.5% of our population, it should be noted that in the present study only hospitalized patients were included, while the reports already detailed also included outpatients. This difference in the frequency of the hospitalized population with CKD is supported by studies that determined that CKD is an independent risk factor for hospitalization and is also the noncommunicable disease with the highest risk of hospitalization (Fresán et al., 2021; Hernández-Galdamez et al., 2020; Soares, Mattos & Raposo, 2020). According to the systematic review by Bajgain et al. (2021), which included studies conducted mostly in Asia, followed by Europe and, in the case of America, only Mexico and the United States, 2.6% of the population with COVID-19 had CKD as a comorbidity.

We decided to include hospitalized patients to control bias, considering moderate-to-severe patients with a clinical severity associated with high mortality (Rodríguez et al., 2020).

The results showed that the prevalence of mortality among patients having CKD as comorbidity *vs* no CKD in the Mexican population was 1.42, while Hernández-Vásquez et al. (2020) reported a mortality risk of 4.37 in the Mexican population until May 2020, with 51,053 confirmed COVID-19 cases. The methodological differences could explain the gap in these results since in the aforementioned study, the results were reported with OR as a measure of association. The differences found may also be due to probable differences in the care and treatment protocols applied to the study population according to the Mexican regulations in force; the previously mentioned study included a population recruited until May 2022, being the guidelines for care by COVID-19 one of the main tools for therapeutic management during that period (Secretaría de Salud, 2020). However, that information did not have evidence-based updates; a situation that is likely to have happened some time later, so that the population served in the following months under the new treatment recommendations reported in July 2020 may have decreased the risk of death in patients with CKD (Gobierno de México, 2020). Given that the database obtained for this study included a population served until September 2020, it is likely that patients will have received a different care and treatment flowchart based on updated scientific evidence by that time. Salinas-Escudero reported a ratio of death prevalence with CKD *vs* no CKD as 6.86 until April (Salinas-Escudero et al., 2020). This difference can be explained by the inclusion of outpatients, which corresponds to 60.72% of this study population, who did not receive the necessary hospital care to avoid fatal outcomes probably in case of typical complications due to the progression of COVID-19. The death prevalence with CKD reported by Salinas and the one reported in the present study differ from the value reported by Bajgain et al. (2021) in a systematic review. They reported a PR of 8.3 in patients with confirmed COVID-19 who had CKD and died compared to those with no CKD. The difference is because the review included mostly studies with an Asian population, in which the major specific comorbidities presented a significant difference between the countries.

Age is significantly associated with mortality in COVID-19. In the present study, it was determined that the population hospitalized with confirmed COVID-19 and aged >65 years had a higher frequency of mortality compared to other age groups. This high mortality is similar to that reported in other studies, where the population >65 years of age has a high frequency of mortality, reaching values of up to 87.1% (Hernández-Vásquez et al., 2020; Martos-Benítez, Soler-Morejón & García-Del Barco, 2021; Parra-Bracamonte et al., 2020). According to these results and findings from other studies, it would be expected to find a higher frequency of patients >65 years of age admitted to the ICU (Nielsson et al., 2014). However, it was the population in the age group of 45–65 years that presented the highest frequency. These results can be explained considering that in March 2020, Mexico reported a total of 4,291 ICU beds and 2,053 mechanical ventilators available in public and private institutions as well as a shortage of medical and nursing personnel (Miranda, 2020). An analysis of the current healthcare context and the high demand for ICU beds as well as personnel and medical resources led to the proposal of certain criteria or triage protocols to determine which patients will be admitted to the ICU (González-Castro et al., 2020; Rubio et al., 2020). It is important to note that the health condition of the elderly, due to the aging factor, is associated with an increase in the prevalence of comorbidities and functional deterioration prior to admission to the ICU (Poma et al., 2012). In results reported in studies that assess age, underlying chronic diseases, and functional capacity among other factors such as triage criteria during the COVID-19 pandemic, it was concluded that elderly patients in severe condition are admitted less frequently to the ICU (Escudero-Acha et al., 2020; Segrelles-Calvo et al., 2020). Therefore, age could be considered as a limiting factor for admission to the ICU.

We expected to find that patients with CKD had a risk factor for admission to the ICU; however, we found that this comorbidity was a protective factor for admission to the ICU. It is considered that this finding corresponds to a possible methodological error related to the decision to admit patients with CKD to the ICU. According to previous studies, it is known that having CKD is a high predictor of mortality (Burhamah et al., 2021), therefore, by increasing the risk of death and representing a greater risk of clinical deterioration (Cheng et al., 2020), it is likely that the health personnel in charge of making the decision as to which type of patient was or was not admitted to the ICU could have decreased the admission of patients with CKD to the ICU. In addition, for the same reasons stated above, in which due to the high number of cases of patients with COVID-19 and the saturation of health services, there were not enough staff and medical facilities for the treatment of patients, so they opted for screening and clinical assessment prior to admission to the ICU.

In the present study, an association was found between chronic conditions and mortality in confirmed COVID-19 cases. According to the results obtained by analyzing the occurrence of CKD with another comorbidity, the death prevalence in those patients with CKD and DM was 1.64 times the probability of dying compared to patients who did not have any other comorbidity. This finding is based on the fact that one of the most common complications of DM is CKD, and patients frequently have both conditions; therefore, their prognoses are closely related (Verner Codoceo, 2010). Furthermore, DM and CKD increase the morbidity and mortality from cardiovascular events (Lou Arnal et al., 2010; Santamaría Olmo & Gorostidi Pérez, 2013). Precisely, the second association with the highest probability of death involves CKD and hypertension, with a prevalence of 1.65. The result is due to the increase in blood pressure that is associated with the progression of CKD owing to the increase in systemic blood pressure in renal microvascularization and the presence of proteinuria, with hypertension being the main cause of the incidence for renal disease (Noboa et al., 2012; Santamaría Olmo & Gorostidi Pérez, 2013; Torres Rondón et al., 2017). Hypertension leads to endothelial dysfunction that can significantly increase progression from moderate to severe COVID-19 (Peña et al., 2021). Finally, it was observed that patients with CKD, DM, and hypertension had a higher death prevalence compared to those with no comorbidity. These diseases lead to chronic inflammatory processes that promote a dysregulated immune response against infection by COVID-19, with a subsequent hyperinflammatory state, activation, and endothelial dysfunction. In addition to increasing the susceptibility to infections, pneumonia is one of the most frequent respiratory complications in COVID-19 (Callender et al., 2020; Juan Carlos Flores, 2010; Noboa et al., 2012; Peña et al., 2021). It should be noted that DM and hypertension are considered two of the three main risk factors for death in COVID-19 (Peña et al., 2021). Plasencia-Urizarri, Aguilera-Rodríguez & Almaguer-Mederos (2020) concluded that CKD, hypertension, and DM constitute the comorbidities with the highest risk of severe clinical presentation in patients with COVID-19.

Limitations of the present study: The database used is secondary in nature and we were unable to evaluate other variables such as difference in treatments, severity of COVID-19, laboratory data, and control of chronic diseases. Hence, we should be cautious while interpreting the findings. In addition, we acknowledge Berkson’s bias, in generalizing these data to Mexican hospitalized patients with CKD and COVID-19. Finally, in relation to the result obtained where CKD would be a protective factor for ICU, it can be pointed out that there could possibly be a survival bias with respect to the ICU outcome.

The study concludes that there is an association between CKD and mortality in patients with a confirmed COVID-19 diagnosis. The results show a high frequency and prevalence of mortality in patients with CKD, a finding supported by other studies conducted in Mexico with results similar to those obtained in the present research. Likewise, an association was found between chronic conditions and mortality in confirmed COVID-19 cases.

## Supplemental Information

10.7717/peerj.13437/supp-1 Supplemental Information 1 Codebook: CKD and COVID-19. ckd, Chronic kidney disease; ckd_dm, Chronic Kidney Disease and Diabetes Mellitus; ckd_dm_hta, Chronic Kidney Disease; Diabetes Mellitus and Hypertesion; ckd_hta, Chronic Kidney Disease and Hypertension; copd, Chronic Obstructive Pulmonary Disease; dm, Diabetes Mellitus; hta, Hypertension; icu, Intensive Care Unit; id, Record ID.

10.7717/peerj.13437/supp-2 Supplemental Information 2 Data: CKD and COVID-19. ckd, Chronic kidney disease; ckd_dm, Chronic Kidney Disease and Diabetes Mellitus; ckd_dm_hta, Chronic Kidney Disease; Diabetes Mellitus and Hypertesion; ckd_hta, Chronic Kidney Disease and Hypertension; copd, Chronic Obstructive Pulmonary Disease; dm, Diabetes Mellitus; hta, Hypertension; icu, Intensive Care Unit; id, Record ID.

## Additional Information and Declarations

### Data Availability

The following information was supplied regarding data availability: The raw data is available in the Supplemental Files.
