# Supplementary material for: Association between chronic kidney disease and mortality in patients with a confirmed COVID-19 diagnosis
Source: PeerJ. 2022 Jun 14;10:e13437. doi: 10.7717/peerj.13437 (PMC9205307; doi:10.7717/peerj.13437)
Supplement: Supplemental Information 1 — ckd, Chronic kidney disease; ckd_dm, Chronic Kidney Disease and Diabetes Mellitus; ckd_dm_hta, Chronic Kidney Disease; Diabetes Mellitus and Hypertesion; ckd_hta, Chronic Kidney Disease and Hypertension; copd, Chronic Obstructive Pulmonary Disease; dm, Diabetes Mellitus; hta, Hypertension; icu, Intensive Care Unit; id, Record ID. [file peerj-10-13437-s001.docx]

**Association between Chronic Kidney Disease and Mortality in Patients with a Confirmed COVID-19 Diagnosis**

| **Variable**  (Variable Name) | **Define** | **Codebooks** | **Reference Group**  Omitted in the regression model |
| --- | --- | --- | --- |
| age | Age group | 1: 18 – 45 years  2: 45 – 65 years  3: Over 65 years | 18 – 45 years |
| cardiovascular | Cardiosvacular disease | 0: No  1: Yes | No |
| ckd | Chronic kidney disease (CKD) | 0: No  1: Yes | No |
| ckd_dm | Chronic Kidney Disease and Diabetes Mellitus | 0: No  1: Yes | No |
| ckd_dm_hta | Chronic Kidney Disease, Diabetes Mellitus and Hypertesion | 0: No  1: Yes | No |
| ckd_hta | Chronic Kidney Disease and Hypertension | 0: No  1: Yes | No |
| copd | Chronic Obstructive Pulmonary Disease (COPD) | 0: No  1: Yes | No |
| died | Died | 0: No  1: Yes | No |
| dm | Diabetes Mellitus | 0: No  1: Yes | No |
| hta | Hypertension | 0: No  1: Yes | No |
| icu | Intensive Care Unit (ICU) | 0: No  1: Yes | No |
| id | Record ID |  |  |
| inmmunosuppression | Inmmunosuppression | 0: No  1: Yes | No |
| obesity | Obesity | 0: No  1: Yes | No |
| pneumonia | Pneumonia | 0: No  1: Yes | No |
| sex | Sex | 0: Male  1: Female | Male |
| smoking | Smoking | 0: No  1: Yes | No |
